# Challenges and Outcomes of the First Stem Cell Transplant Program in Tanzania, East Africa

**DOI:** 10.1155/2024/1937419

**Published:** 2024-03-16

**Authors:** Stella Rwezaula, Mbonea Yonazi, Amey Panchal, Ashish Dhoot, Jemy Mathew, Sonu Tony, Sandeep Rao, Peter Muhoka, Samira Mahfudh, Neema Budodi, Mabula Kasubi, Flora Ndobho, Helena Kakumbula, Koga Luhulla, Linda Kapesa, Heri Tungaraza, Sarah Nyagabona, Agnes Shayo, Felister Seleki, Janeth Mtenga, Khadija Mwamtemi, Musa Suko, Isaac Mbughi, Mariana Shirima, Alfayo Mkisi, Rahma Ally, Malselina Kyaruzi, Else Arola Myaka, Johari Matiku, Mariam Nyamwaira, Saranya Nair, Aswathy Asokan, Goutham kumar, Raj Badavath, Hedwiga Swai, Lawrence Museru, B. S. Ajaikumar, Deogratius Beda, Sachin Jadhav

**Affiliations:** ^1^Muhimbili National Hospital, Dar es Salaam, Tanzania; ^2^Healthcare Global Enterprises Limited, Bangalore, India

## Abstract

**Introduction:**

Due to the significant resources involved in creating HSCT programs there is a significant disparity in the availability of this treatment modality between the developed and developing countries. This manuscript details the process and the outcomes of the first HSCT program in East Africa which was started at Muhimbili National Hospital (MNH) in Dar-es-Salaam, Tanzania.

**Materials and Methods:**

Information and data were collected on the processes which had been implemented for starting the HSCT program at MNH. The details of the collaborations, training, infrastructure development, and acquisition of the biomedical equipment, as well as the actual process for HSCT, as well as the outcomes of treatment are described. *Observations*. The project has been detailed in 4 stages for ease of description: Stage 1: Preparatory work which was performed by the Government of Tanzania, as well as the administrators and clinicians from MNH (July 2017-September 2021). Stage 2: Exploratory gap analysis by the teams from MNH and International Haematology Consortium of HCG Hospital, India (HCG-IHC) in October 2021. Stage 3: Activities for closure of gaps (November 2021). Stage 4: Stem Cell Transplantation Camps (November 2021 to March 2022). 11 peripheral blood stem cell transplants were done in two camps, November 2021 (5 patients), and February 2022 (6 patients). 10 patients underwent autologous peripheral blood stem cell transplantation for multiple myeloma and 1 for lymphoma. The median duration of hospital stay was 19 ± 6 days. The median time for neutrophil engraftment, it was on 8.8 ± 0.8 days, and for platelet engraftment was 9.6 ± 2.4 days. Progression-free survival was 100%, and there was no mortality.

**Conclusion:**

Commonalities in the socioeconomic challenges in developing countries can be leveraged to create robust HSCT programs in other developing countries.

## 1. Introduction

Hematopoietic stem cell transplantation (HSCT) is the standard of care for several hematological malignancies, and certain congenital or acquired disorders [1].

The availability of stem cell transplant programs has been impacted by differences in resource availability between industrialized and developing nations [2]. At the time of writing this manuscript, HSCT programs in Africa are only available in the countries of South Africa and Nigeria, which are in west and southern Africa. However, HSCT is available to only 3% of the population on that continent [2]. Tanzania, an East African country, has lacked a robust program for HSCT, and thus benign disorders, such as sickle cell anemia, aplastic anemia as well as hematologic malignancies cannot be appropriately treated there [3, 4]. The impact of this on patient outcomes is evidenced by the 48% mortality rate among patients diagnosed with severe aplastic anemia [4]. Similarly, the approximate annual incidence of leukemia in Tanzania is 1898 patients, and a significant number of these will require HSCT, as part of their treatment algorithm [3]. There is thus a clear need for establishing HSCT programs in this part of the world [3, 4].

This manuscript details the process and the outcomes of the first HSCT program in East Africa which was started at Muhimbili National Hospital (MNH) in Dar-es-Salaam, Tanzania.

## 2. Materials and Methods

Information and data were collected on the processes which had been implemented for starting the HSCT program at MNH. The details of the collaborations, training, infrastructure development, and acquisition of the biomedical equipment, as well as the actual process for HSCT, as well as the outcomes of treatment are described.

### 2.1. The Project Has Been Detailed in 4 Stages for Ease of Description

#### 2.1.1. Stage 1: Preparatory Work Which Was Performed by the Government of Tanzania, as well as the Administrators and Clinicians from MNH

Over the last few years, there have been active efforts by Muhimbili National Hospital (MNH) in Dar-es-Salaam, Tanzania, to enhance the availability of appropriate treatment for hematologic disorders. This has been done through the development of a sickle cell programme as well as with training for doctors and nurses, by forming partnerships with centres performing HSCT in India. The details of this are as given in Table 1.

#### 2.1.2. Stage 2: Exploratory Gap Analysis by the Teams from MNH and HCG-IHC

A joint team of MNH (Muhimbili National Hospital) and HCG-IHC conducted an initial site visit at MNH, Upanga, and Mloganzila campuses, in October 2021 to perform a gap analysis as per the Assessment Tool given in Table 2. The infrastructure and the biomedical equipments were divided between MNH, Mloganzila campus and MNH, Upanga campus, which were 30 kilometres and about 1 hour apart by road. The details of these are mentioned in Table 3.

#### 2.1.3. Stage 3: Activities for Closure of Gaps

The gaps were addressed in two phases, as given in Tables 3 and 4.

- The first phase, which was the online phase was from November 1^st^ 2021 to November 20^th^ 2021 and was conducted via WhatsApp™ Groups and Zoom™ videoconferencing.
- The second phase, which was on-site, was conducted at both the MNH facilities in Upanga and Mloganzila, between 22^nd^ November to 25^th^ November 2021.

The activities for protocol transfer and adaptation, online training, hands-on training, and policymaking were divided between these two phases. Four teams were created for this purpose. Each team was headed by a member of HCG-IHC.

#### 2.1.4. Stage 4: Stem Cell Transplantation Camps

As of now, two “HSCT Camps” have been conducted. The first camp was between 23^rd^ November and 17^th^ December, 2021, when 5 patients underwent autologous stem cell transplantation. The second camp was conducted between 20^th^ February and 10^th^ March 2022, when 6 patients underwent autologous stem cell transplantation.

The pretransplant workup and consenting were performed by the MNH team in Tanzania and were approved by the HCG-IHC team via videoconferencing. The conditioning regimen was prepared by the HCG-IHC team and was shared with the MNH team via e-mail. The scheduling of apheresis was done such that two patients could be apheresed per day. G-CSF for mobilization was thus started in a staggered manner by the MNH team. The HCG-IHC team of 4 members (program director, apheresis director, one transplant nurse and one attending physician) reached Tanzania one day before the first apheresis was due, along with the essential drugs and stem cell apheresis kits and consumables.

The stem cell harvest, CD34+ cell enumeration, conditioning chemotherapy, stem cell infusion, and the posttransplant clinical and nursing care were done jointly by the HGC-IHC and MNH teams.

### 2.2. Observations

#### 2.2.1. Transplantation Details

Injection Filgrastim (5 mcg/kg twice daily for 4 days and 10 mcg/kg on the morning of the apheresis) was used for mobilization in all patients. However, due to an assumed difficulty in the pre-harvest enumeration of CD34+ cells in the peripheral blood as well as a limitation in the number of apheresis kits and consumables, injection of Plerixafor (24 mg) via subcutaneous route was given to all the patients to prevent harvest failures in the first camp. However, in the second HSCT camp, reliable preharvest CD34+ cell enumeration could be performed from the peripheral blood. Hence, plerixafor was required for only one patient.

Similarly, to ensure adequate venous access for apheresis, all the 5 patients in the first camp underwent insertion of a central venous catheter in the femoral vein. A double-lumen dialysis catheter was used for this. However, during the second camp, peripheral blood apheresis could be done successfully with peripheral venous access in all but one of the six patients.

Peripheral blood stem cells were harvested through apheresis performed with the Spectra Optia™ cell separator (TERUMOBCT™, Colorado, USA) via continuous mononuclear cell collection procedure as per the manufacturer's instructions. A total of 4 total blood volumes (TBV) were processed for each patient over four-five hours with a target of getting a minimum dosage of 5 × 10^6^/kg CD34+ cells. All the patients tolerated the procedure for the full 4 TBVs without any adverse reactions and had an adequate stem cell dosage harvested in a single setting, except for one patient who required apheresis for two days.

The adequacy of the harvest was measured with CD34+ cell enumeration via flow cytometry and was cross-checked with the mononuclear cell count, which was performed by Leishman staining of the harvest smear. Gram staining and microbiologic culture of all the stem cell products were negative for microbiologic contamination.

Key challenges for the stem cell harvest and engraftment are noted in Table 5.

As mentioned earlier, up to the writing of this manuscript, two SCT camps have been conducted for 11 patients. The first camp involved autologous SCT for 5 patients with multiple myeloma, and the second one was conducted after 3 months for 5 myeloma and 1 patient with relapsed Hodgkin lymphoma. The stem cell products for the myeloma patients were stored at 2–6°C for the next 24–48 hours before infusion. For the Hodgkin lymphoma patient, stem cells were cryopreserved and stored in liquid nitrogen using the controlled rate freezer at −150°C. The peripheral blood stem cell harvest was done on the Upanga campus, and the patients and the stem cell products were transported to the SCT Unit was at MNH Mloganzila, which was 30 km away. This transport of patients and stem cells by road thus took about 1 hour. One test run of a noncryopreserved packed red cell unit and a cryopreserved buffy coat, each was conducted to validate the temperature control during transport.

None of the patients had any adverse transfusion reactions during the stem cell infusions.

The clinical details of the patients are given in Table 6. Overall, 10 patients with multiple myeloma and 1 patient with relapsed classical Hodgkin lymphoma (CHL) underwent autologous SCT. 3 patients (30%) had relapsed multiple myeloma. M : F ratio was 3 : 5, the median age was 43.6 years with a range of 31–65 years. The international staging system and cytogenetic risk assessment were not available for the patients. Imaging studies done for the CHL patient revealed that the patient was in CR2. 8 patients were conditioned with high-dose melphalan (200 mg/m^2^), and 2 patients were conditioned with a melphalan dose of 140 mg/m^2^, one due to age >60 years and the other due to a prior history of renal failure requiring dialysis. The Hodgkin lymphoma patient received conditioning regimen with BCNU, cytarabine, etoposide, and melphalan (BEAM regimen).

All patients underwent cryotherapy as part of standard prophylaxis for oral mucositis. No gut decontamination was performed, and no antibiotics were used prophylactically.

A total of 8 units of SDP were required for all the patients during their cytopenic period with an average requirement of 0.72 bag during the neutropenic phase. A total of 15 units of granulocyte transfusions were required for the management of febrile neutropenia with an average requirement of 1.36 units during the neutropenic phase.

**Figure 1:**
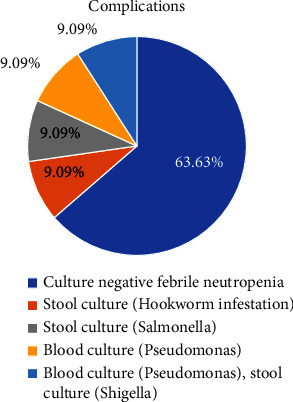
Major post-transplant complications.

Major post-transplant complications were bacteremia (2), culture-negative febrile neutropenia (7), and gastro-intestinal infection (3) (Figure 1).

The median duration of the hospital stay was 19 ± 6 days. The median neutrophil engraftment was on 8.8 ± 0.8 days, and median platelet engraftment was 9.6 ± 2.4 days. 1 patient suffered from salmonella enteritis and was treated with meropenem as per the sensitivity report. Another patient had shigella enteritis and was treated with 3^rd^ generation cephalosporin (Cefixime) and fluoroquinolone (Levofloxacin) as per the culture and sensitivity report.

The median observation period for survivors was 75.45 days post-transplant (126 days–33 days), and these patients were followed up for a median of 552 days. The progression-free rate was 100% over a median observation period of 75.45 days. The cumulative incidence of relapse was nil (0%) and transplant-related mortality (TRM)/non-transplant-related mortality (NRM) in the first 100 days for 5 patients was nil (0%).

## 3. Conclusions

Performing stem cell transplantation in a developing country has its own set of challenges. Standardization of services, starting from the infrastructure to the clinical management of patients was required so that the standard of care was maintained. However, due to similar challenges in terms of the socioeconomic situation, as well as the availability or the lack thereof, of medical infrastructure, biomedical equipment, drugs and consumables, medical and nursing know-how, and standard operating procedures, HCG-IHC, India, could collaborate with MNH, Tanzania, in an efficient manner and clinically relevant manner. We believe that detailing our experience in creating the first stem cell transplant program in East Africa could benefit other centers who are desirous of doing the same in LMICs. This could lead to improved access to HSCT in the developing world, while maintaining acceptable clinical outcomes.

## Figures and Tables

**Table 1:** Preparatory work.

Time period	Activity	People involved	Location	Duration/details
July 2017	Clinical training on HSCT	Six people (one oncologist, one haematologist, one pharmacist, and three nurses)	BLK hospital, India	3 months

August 2018	Clinical training on HSCT	One haematologist	Apollo hospital, Chennai, India	1 month
	Clinical training on HSCT	One oncologist	Apollo hospital, Bangalore, India	1 month
	Clinical training on HSCT	One oncologist	Apollo hospital, Mumbai, India	1 month

June 2019	Planning committee was created	13 people (two haematologists, two hospital administrators, one microbiologist, three lab scientists, one biomedical engineer, one civil engineer, one mechanical engineer, one procurement officer and director finance)	MNH	5 months
				(1) Gather information on procuring medications and consumables
				(2) The director of finance was responsible for conducting cost analysis for one procedure and advised the government on the requisites required for the stem cell transplant unit
				(3) The engineers renovated the HSCT rooms in MNH, mloganzila and ensured that the stem cell transplant unit is HEPAfiltered
				(4) The biomedical department underwent training on the equipments which would be used during the stem cell transplant

December 2019	Sanctioning of financial resources: Tsh 6.2 billion (∼$2.5 million)	Government of Tanzania	(1) Procuring equipment for the cell processing unit, renovation of the of the day care chemotherapy facility and the stem cell transplant unit	
			(2) In-house training of 30 nurses on HSCT by a team from USA	

September 2021	Collaboration was created between MNH and the international haematology consortium, based in HealthCare global (HCG-IHC) hospital in Bangalore, India			Detail the roles, responsibilities and the operational methodology for creating an HSCT program at MNH

**Table 2:** Assessment tool.

*Infrastructure of the stem cell transplant unit*
(i) HEPA-filtered rooms with attached washroom
(ii) Clean and dirty utility areas
(iii) Biomedical equipment: infusion and syringe pumps, cardiac monitors, medication carts, crash cart, computers for accessing the hospital and laboratory information systems

*Apheresis facility*
(i)Apheresis equipment × 2
(ii) Appropriate stem cell harvest kit
(iii) ACD bags and other consumables

*Stem cell laboratory for CD34+ cell enumeration and stem cell storage*
(i) Flow cytometer with updated software
(ii) Reagents for standardization of CD34+ cell enumeration
(iii) CD34+ cell enumeration beads and reagents
(iv) Controlled rate freezer with liquid nitrogen storage tank
(v) Liquid nitrogen shipper
(vi) Liquid nitrogen supply
(vii) Trained laboratory technologist

*Team*
(i) Attending physicians
(ii) Associate physicians
(iii) Registered nurses
(iv) Clinical pharmacist
(v) Housekeeping staff

*Policies and protocols*
(i) Standard operating protocols (SOP) for pretransplant workup and consent
(ii) Nursing policies and SOPs
(iii) Infection control policy
(iv) Febrile neutropenia management policy
(v) Central venous access insertion and maintenance bundle
(vi) Transfusion policy
(vii)SOPs for peripheral blood stem cell harvest, storage, transport and infusion

*Drugs*
(i) Antibiotics and antifungals
(ii) G-CSF
(iii) Plerixafor
(iv) Chemotherapeutic drugs

*Allied departments*
(i) Intensive care unit
(ii) Transfusion medicine service with availability of blood components
(iii) Laboratory with a quality management plan for hematologic and biochemistry tests and for blood culture and sensitivity
(iv) Dietetics department with hygiene control and pest control
(v) Radiology department with the ability to insert central venous catheters

**Table 3:** Result of the initial gap analysis.

Available	Not available
*Stem cell transplant unit at MNH, mloganzila campus*	
(i) 6 hepa-filtered rooms	(i) Infusion and syringe pumps
	(ii) Biomedical equipments including, cardiac monitors, medication carts, crash cart, computers for accessing the hospital and laboratory information systems

*Apheresis facility at MNH, upanga campus*	
(i) Apheresis equipment: Spectra Optia™ cell separator (TERUMOBCT™, Colorado, USA) × 2	(i) Optia™ stem cell harvest kit
	(ii) ACD bags compatible with Optia™

*Stem cell laboratory at MNH, muhimbili campus*	
(i) BD FACS canto II (ii) Controlled rate freezer with liquid nitrogen storage tank	(i) Reagents for standardization of CD34+ cell enumeration
	(ii) CD34+ cell enumeration beads and reagents
	(iii) Software for CD34+ cell enumeration
	(iv) Liquid nitrogen shipper
	(v) Liquid nitrogen supply

*Team*	
(i) 5 attending physicians and 3 registered nurses (RN), 1 pharmacist, 3 lab scientists and 1 microbiologist had previously undergone training in the clinical management of HSCT patients	(i) Trained associate physicians
	(ii) Larger team of trained RN
	(iii) Trained housekeeping staff

*Policies and protocols*	
(i) Antibiotics and antifungals	(i) SOPs for pretransplant workup and consent
	(ii) Nursing policies and SOPs
	(iii) Infection control policy
	(iv) Febrile neutropenia management policy
	(v) Central venous access insertion and maintenance bundle
	(vi) Transfusion policy
	(vii) SOPs for peripheral blood stem cell harvest, storage, transport and infusion
	(viii) Non-availability of critical drugs such as melphalan, plerixafor, etc

**Table 4:** Action plan for closing the gaps.

Teams	Members	Online phase	On-site phase
Supervisory team	HCG, India	(i) Overall planning and coordination of the SCT program	(i) Febrile neutropenia policy
	(i) BMT program director		
	MNH, Tanzania		
	(i) Head of department of haematology and BMT	(i) Importing drugs, reagents and consumables	(i) Coordination between collaborating departments such as the clinical laboratory, transfusion medicine, dietetics, etc
	(ii) Senior consultant		
	(iii) Head of nursing		
	(iv) Clinical pharmacologist		
	(v) Head of stem cell laboratory		

Nursing	HCG, India	(i) Hand hygiene	(i) Rounding
	(i) Head of Haematology-BMT nursing	(ii) Sterile reconstitution of injectable drugs	(ii) Clinical nursing in a SCT unit
	(ii) Nursing educator, haematology-BMT	(iii) PICC line sampling and dressing	
	(iii) Quality manager, haematology-BMT	(iv) Nursing monitoring	
	MNH, Tanzania		
	(i) Nursing head, haematology		
	(ii) Nursing team, haematology		

Physicians team	HCG, India	(i) Overview of SCT	(i) Patient monitoring and documentation
	(i) BMT program director	(ii) Rounding	
	(ii) One attending physician	(iii) Screening patients pretransplant	
	(iii) BMT coordinator	(iv) Evaluation and consenting	
	MNH, Tanzania		
	(i) Head of department of haematology and BMT		
	(ii) All the physicians in Haematology-BMT		

Stem cell laboratory	HCG, India	(i) Apheresis training	(i) Stem cell product labelling, storage and transport
	(i) Director, stem cell laboratory	(ii) CD34+ cell enumeration	
	(ii) Apheresis technologist		
	(iii) Hematopathologist		
	(iv) Technologist, hematopathology laboratory		
	
	MNH, Tanzania		
	(i) Director, stem cell laboratory		
	(ii) Apheresis technologist		
	(iii) Hematopathologist		
	(iv) Technologist, hematopathology laboratory		

**Table 5:** Key challenges for the stem cell harvest and engraftment.

Challenges	Intervention	Result	Future
Assumed difficulty in the pre- harvest enumeration	Inj. PLERIXAFOR 24 mg S/C for all patients during 1^st^ camp	Adequate stem cell collection (except one patient who required 2 days collection)	Camp 2- Reliable CD34 cell enumeration – Inj. PLERIXAFOR required only for 1 patient
Limitation in the number of apheresis kits and consumables			

Venous access	CVC (femoral)- camp 1–5 patients	Apheresis procedure uneventful	Camp 2- CVC required only for 1 patient

Febrile neutropenia management	Inj. Peg G-CSF 6 mg SC weekly from day +3 until engraftment	Early engraftment	

**Table 6:** Clinical details.

UPN	Age	Sex	Disease status pretranspl ant	Date of transplant	CD34 and MNC counts	Mucosi tis details	Infection details	Neutroph il engraftm ent	Platelet engraftm ent	Durati on of inpatie nt stay
1	36	M	CR2	25/11/20	12.3 × 10^6/kg	Grade II	Culture negative	D + 8	D + 8	25
				22						

2	55	M	PR	27/11/20	12 × 10^6/kg	Grade III	Stool culture –hookworm infestation	D + 9	D + 10	24
				22						

3	49	M	CR1	27/11/20	21.4 × 10^6/kg	Grade III	Stool culture – salmonella	D + 9	D + 9	24
				22						

4	34	M	NA	26/11/20	17.17 × 10^6/kg	Grade II	Pseudomo nas aeruginosa bacteremia	D + 8	D + 12	23
				22						

5	46	F	CR2	29/11/20	5.709 × 10^6/kg	Grade II	Culture negative febrile neutropeni a	D + 10	D + 10	22
				22						

6	37	M	CR1	23/02/20	9.6 × 10^6/kg	Grade II	Culture negative	D + 9	D + 11	14
				22						

7	31	F	CR1	23/02/20	9.5 × 10^6/kg	Grade II	Culture negative	D + 8	D + 10	14
				22						

8	65	F	CR1	24/02/20	3.44 × 10^6/kg	Grade II	Blood culture – pseudomo nas aeruginosa stool culture - shigella	D + 9	D + 11	13
				22						

9	45	M	VGPR	24/02/20	12.5 × 10^6/kg	Grade II	Culture negative	D + 8	D + 9	13
				22						

10	38	F	CR1	25/02/20	9.8 × 10^6/kg	Grade II	Culture negative	D + 8	D + 10	12
				22						

11	31	M	CR2	26/02/20	13.63 × 10^6/kg	Grade I	Culture negative	D + 10	D + 12	16
				22						

## Data Availability

The data used to support the findings of this study are available from the corresponding author upon request.
